# A de novo evolved domain improves the cadmium detoxification capacity of limpet metallothioneins

**DOI:** 10.1038/s41598-023-35786-1

**Published:** 2023-06-01

**Authors:** Mario García-Risco, Sara Calatayud, Veronika Pedrini-Martha, Ricard Albalat, Òscar Palacios, Mercè Capdevila, Reinhard Dallinger

**Affiliations:** 1 Present Address: Departament de Química, Facultat de Ciències, Universitat Autònoma de Barcelona, 08193 Cerdanyola del Vallès, Spain; 2 Departament de Genètica, Facultat de Biologia, Microbiologia i Estadística and Institut de Recerca de la Biodiversitat (IRBio), Universitat de Barcelona, Av. Diagonal 643, 08028 Barcelona, Spain; 3 Institute of Zoology, Center of Molecular Biosciences, University of Innsbruck, Technikerstraße 25, 6020 Innsbruck, Austria

**Keywords:** Biochemistry, Ecology, Evolution, Physiology, Zoology

## Abstract

Metallothioneins (MTs) constitute an important family of metal binding proteins. Mollusk MTs, in particular, have been used as model systems to better understand the evolution of their metal binding features and functional adaptation. In the present study two recombinantly produced MTs, LgiMT1 and LgiMT2, and their de novo evolved γ domain, of the marine limpet *Lottia gigantea*, were analyzed by electronic spectroscopy and mass spectrometry. Both MT proteins, as well as their γ domains, exhibit a strong binding specificity for Cd(II), but not for Zn(II) or Cu(I). The LgiMTs’ γ domain renders an M^II^_4_(SCys)_10_ cluster with an increased Cd stoichiometry (binding 4 instead of 3 Cd^2+^ ions), representing a novel structural element in the world of MTs, probably featuring an adamantane 3D structure. This cluster significantly improves the Cd(II)-binding performance of the full length proteins and thus contributes to the particularly high Cd coping capacity observed in free-living limpets.

## Introduction

Interactions between metals and organisms are intrinsic to life, whereby metals constitute ubiquitous and essential components of the biological systems. Some metallic trace elements such as iron (Fe), zinc (Zn) or copper (Cu) are essential micronutrients and trace elements required for the functionalization of numerous enzymes and proteins. Other metals such as lead (Pb), cadmium (Cd) or mercury (Hg) are highly reactive and, even at low concentrations, might displace essential metals from their active binding sites, resulting in very harmful effects. All living beings require, therefore, mechanisms to control the homeostasis of the essential metals as well as to counteract the harmful effects of the non-essential ones. One of these mechanisms is based on the activity of metallothioneins (MTs), a heterogeneous family of metal-binding proteins that have been involved in the physiological control of metals operating as ion reservoirs, metal transporters and/or metal deliverers to target metalloproteins, but also in radical scavenging, oxidative stress protection and anti-apoptotic defense (reviewed in^1,2^). Interestingly, in a same organism, MT isoforms might have different metal preferences or be expressed in different tissues or in response to different stimuli, supporting that MTs perform diverse physiological functions^3,4^, many of them still unknown. After 65 years from their discovery and identification in 1957^5^, MTs still remain enigmatic^6,7^, and a hot topic of research in many scientific areas, from Biology through Chemistry to Environmental Sciences, but also Physics, Medicine, Toxicology and Pharmacology.

MTs are characterized by their low molecular weight and their high percentage of cysteines (Cys) in their amino acid sequences^8^. The high number of Cys provides to these proteins, through their thiol groups, the ability of multiply coordinating a variety of metal ions^1,9^. In their apo form (*i.e.* the demetalated state), MTs lack a defined secondary and tertiary structure, meaning that they only exhibit a well-folded structure in association with metal ions^10^. Moreover, the same MT can be folded in distinct ways depending on the type and the number of metal ions that the peptide is coordinating^11^. Historically, MTs were natively found associated with Zn(II) and/or Cd(II)^12^, but when new MTs were discovered, Cu(I) was also found to be natively bound to some of them^13,14^, already revealing that not all MTs show the same abilities to bind Zn(II), Cd(II) or Cu(I) ions^4,15,16^. The number of Cys and their position in the amino acid sequence, together with the nature of some non-coordinating amino acids, determine the metal-binding preference as well as the metal-binding capacity of the MTs^17–19^. These structural features define whether an MT will natively bind divalent -Zn(II) and/or Cd(II)- or monovalent -Cu(I)- metal ions, and the number of ions that it can load^4^.

Sequence analyses have revealed that in MTs, Cys residues are arranged in distinctive motifs (i.e. CxC, CC and CCC) whose number and distribution define the MT domains: α domains with 11–12 Cys, and β domains with 9 Cys. In this modular structure, each domain is able to independently form different kinds of metallic clusters depending on the number and disposition of their Cys residues^1,20^. To date, two types of metal clusters have been described in mammalian MTs binding Zn(II) or Cd(II), the M^II^_3_(SCys)_9_ clusters for the β domains, and the M^II^_4_(SCys)_11_ clusters for the α domains^21,22^. Both types of metal-Cys aggregates are characteristic of vertebrate MTs^23^ but have also been found in species of other animal phyla. In invertebrates, most three-dimensional structures solved by NMR are from gastropod MTs^24^, which mostly are bidominial proteins made of a β3 domain (Cx_n_Cx_n_[CxC]x_n_[CxC]x_n_[CxC]x_n_C) at the N-terminal region, and a β1 domain ([CxC]x_n_[CxC]x_n_Cx_n_[CxC]x_n_[CxC]) at the C-terminal end (i.e. β3/β1-MTs)^25^. These gastropod domains can independently bind 3 divalent metal ions forming M^II^_3_(SCys)_9_ clusters. We have recently identified two new gastropod MTs in *Lottia gigantea*, a marine mollusk of the Patellogastropoda clade^25^. Surprisingly, *L. gigantea* MTs (LgiMT1 and LgiMT2) exhibited a domain organization diverging from the prototypical gastropod β3/β1-MT, since their N-terminal domains were different from any other MT domain hitherto known and thus representing an evolutionary innovation. The novel N-terminal domain, which was named γ domain, comprises ten Cys arranged in five CC duplets. Hence, the structure of *L. gigantea* MTs is of γ/β1 domains. In the present work, the metal-binding abilities of both *L. gigantea* MTs and of the new γ domain were thoroughly characterized after their recombinant production in the presence of Zn(II), Cd(II) and Cu(II) metal ions. Purified metal-protein complexes have been characterized by UV–Vis and circular dichroism spectroscopies and mass spectrometry, revealing the metal-binding features of the analyzed proteins, as well as the functional autonomy and structural independence of the γ domain. Interestingly, the present results show that the γ domain forms an M^II^_4_(SCys)_10_ cluster probably with an adamantane 3D structure, which represents a new structural motif in the world of MTs. Nevertheless, the novel γ-domain of MTs from *L. gigantea* represents an evolutionary step forward by improving the Cd-binding performance of these proteins and thus the Cd detoxification capacity of this common limpet species.

## Materials and methods

### Production and purification of recombinant metal-MT complexes

Production and purification of recombinant metal-MT complexes were performed as described elsewhere^25^. Our methodological approach was based on compelling evidence that heterologous productions of MTs yield metal-MT complexes structurally and functionally equivalent to those isolated from native sources^17,26–28^. In brief, synthetic cDNAs codifying LgiMT1, LgiMT2 and the γ domain of LgiMT2 (from Met1 to Gln45) were provided by Synbiotech (Monmouth Junction, NJ, USA) and cloned in the pGEX-4 T-1 expression vector (GE Healthcare, Chicago, IL, USA) in order to produce GST-MT fusion proteins. Recombinant metal-MT complexes were produced in *E. coli* BL21 cells transformed with the corresponding recombinant plasmids, grown in Luria–Bertani (LB) medium with 100 μg/mL ampicillin, and induced with 100 μM isopropyl-β-D-thiogalactopyranoside for 3 h. After the first 30 min of induction, cultures were supplemented with ZnCl_2_ (300 μM), CdCl_2_ (300 μM), or CuSO_4_ (500 μM) in order to generate metal-MT complexes. Recombinant GST-MT fusion proteins were purified from soluble protein extracts with glutathione sepharose beads (GE Healthcare) and digested with thrombin (GE Healthcare, 25U/L of culture), thus enabling separation of the metal-MT complexes from the GST that remained bound to the sepharose matrix. Here, it should be noticed that recombinant MTs produced in this way contain two additional amino acids (GS) at their N-term after purification. It has been demonstrated that these additional amino acids do not alter the metal-binding abilities of the recombinant MTs^28,29^. The eluted metal-MT preparations were concentrated with a 3-kDa Centripep Low Concentrator (Amicon, Millipore, MA, USA) and fractionated on a Superdex-75 FPLC column (GE Healthcare). The protein-containing fractions, identified by their absorbance at 254 nm, were pooled and stored at − 80 °C until use.

### Metal determination and protein quantification of LgiMT1, LgiMT2 and γLgiMT2 preparations

Solutions containing the metal-MT complexes purified from the bacterial production were diluted with HNO_3_ 1% (v/v). Their S, Cu, Zn and Cd content was determined by means of inductively coupled plasma-atomic emission spectrometry (ICP-AES). A Perkin-Elmer Optima 4300DV (Waltham, USA) spectrometer performed the element quantification (S, Cu, Zn and Cd) of the samples at the correct wavelength (S, 182.04 nm; Zn, 213.86 nm; Cd, 228.80 nm; Cu, 324.80 nm) under conventional conditions^30^. Protein concentration was calculated based on the S concentration obtained by ICP-AES, assuming that all the sulfur measured comes from peptides’ Cys and methionine (Met) residues.

### Metal-LgiMT1, LgiMT2 and γLgiMT2 species determination

Positive ion electrospray ionization time-of-flight mass spectrometry (ESI-TOF MS) was performed on the samples obtained from the purification process, previously equilibrated with 20 mM Tris–HCl at pH 7. A Micro Tof-Q Instrument (Bruker Daltonics Gmbh, Germany), calibrated with ESI-L Low Concentration Tuning Mix (Agilent Technologies, USA) and interfaced to a Series 1100 HPLC pump (Agilent Technologies) was used to determine the molecular weight of the metal-MT species. This process was performed under physiologic (pH 7) and acidic (pH 2.4) conditions. Under acidic conditions, Zn- and Cd-loaded forms exchange the metal by protons and apo-form’s molecular weight is determined^31^. The assay conditions were as follows: 20 µL of sample injected at 30–50 µL·min^-1^ at 3.5–5.0 kV capillary-counter voltage, 90–110 °C of desolvation temperature, and dry gas at 6 L·min^-1^. Spectra were recorded between a *m/z* range from 800 to 3000. The liquid carrier for native conditions was a 90:10 mixture of 15 mM ammonium acetate and acetonitrile at pH 7.0, while acidic conditions was a 95:5 mixture of formic acid and acetonitrile at pH 2.4.

### Spectroscopic characterization (CD and UV–Vis)

UV–Vis spectrometry was performed to the samples by means of a HP-8453 diode array UV–visible spectrophotometer (Hewlett Packard, USA) to assess their absorption features. Moreover, to investigate charge-transfer transitions of the metal-LgiMT1, -LgiMT2 and -γLgiMT2 complexes, CD determinations were performed in the UV–Vis range (200–500 nm) using a Jasco spectropolarimeter (J-715; Jasco Inc., USA) interfaced to a computer (J700 software). Raw data obtained from these experiments was processed with GRAMS32 software (GRAMS/AI v.7.02; USA).

## Results and discussion

### Recombinant production of metal-MT complexes of *Lottia gigantea* metallothioneins

**Figure 1:**
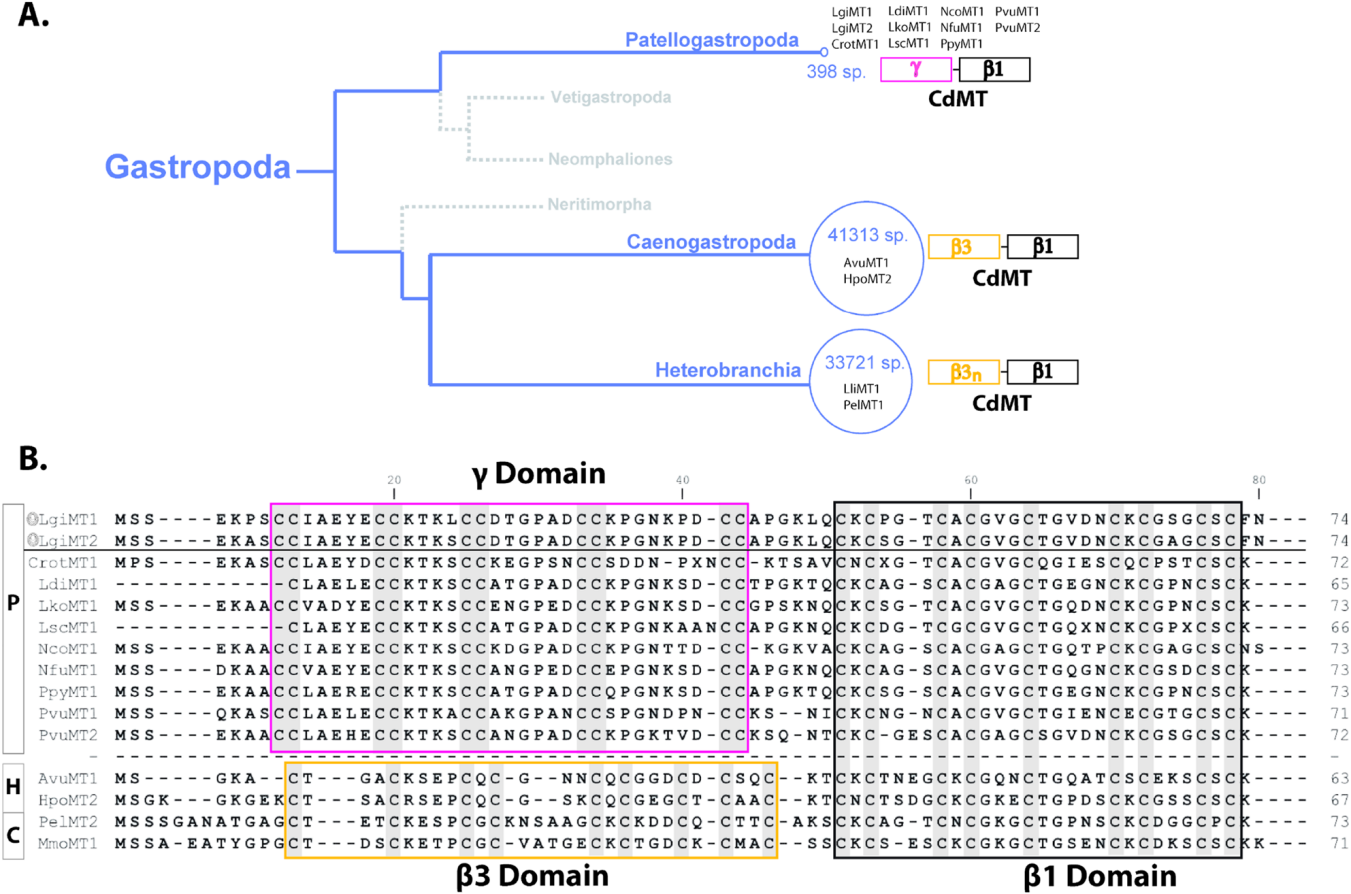
(**A**) Simplified phylogenetic tree of the Gastropoda class. Blue branches depict Patellogastropoda with 398 species and the sister clades Caenogastropoda (with 41,313 species) and Heterobranchia (with 33,721 species) comprising, together, the vast majority of the species so far described in Gastropoda. Alongside the branch of Patellogastropoda the MTs so far reported for this group are listed^25^, and also indicated is the respective MT organization in a bi-domain structure with a γ and β1 domain. For the other two clades, Caenogastropoda and Heterobranchia, only two representative MTs are reported here with their bi-domain organization of a β3/β1 and β3n/β1 structure, respectively. (**B**) Amino acid alignment of *L. gigantea* MTs (LgiMT1 and LgiMT2) with other Patellogastropoda MTs (P) – *Cellana rota* (CrotMT1), *Lottia digitalis* (LdiMT1), *Lottia kogamogai* (LkoMT1), *Lottia scutum* (LscMT1), *Nacella concinna* (NcoMT1), *Nipponacmea fuscoviridis* (NfuMT1), *Patelloida pygmaea* (PpyMT1), *Patella vulgata* (PvuMT1 and PvuMT2) – and two species of Heterobranchia (H) – *Arion vulgaris* (AvuMT1) and *Helix pomatia* (HpoMT2) – and two Caenogastropoda species (C) – *Pomatias elegans* (PelMT2) and *Marseniopsis mollis* (MmoMT1) – Cysteines defining the MT domains are highlighted with a grey background. The γ domain is shown within a pink frame box, the β3 domain within an orange frame box, and the shared β1 domain with a black frame box.

As already mentioned above, two new MTs, LgiMT1 (accession number QHN12717) and LgiMT2 (accession number QHN12718), had been identified in the marine gastropod *L. gigantea*^25^. Both MTs were 74 amino acid long with 19 Cys (25.7%), only differing at positions 6, 20, and 49: Pro, Lys, and Pro residues in LgiMT1, and Ala, Ser, and Ser in LgiMT2 (Fig. 1). The 19 Cys were organized in two domains: a novel γ domain at the N-terminal end with ten Cys residues arranged in five CC pairs (CCx_5_CCx_4_CCx_6_CCx_7_CC), which was connected by a linker of six amino acids to an archetypal 9-Cys β1 domain ([CxC]x_3_[CxC]x_3_Cx_5_[CxC]x_3_[CxC]) at the C-terminal region (Fig. 1). Significantly, the MTs of eight additional Patellogastropoda species shared this γ/β1 organization, meaning that the γ domain likely was a successful structural innovation of this whole gastropod lineage^25^.

**Figure 2:**
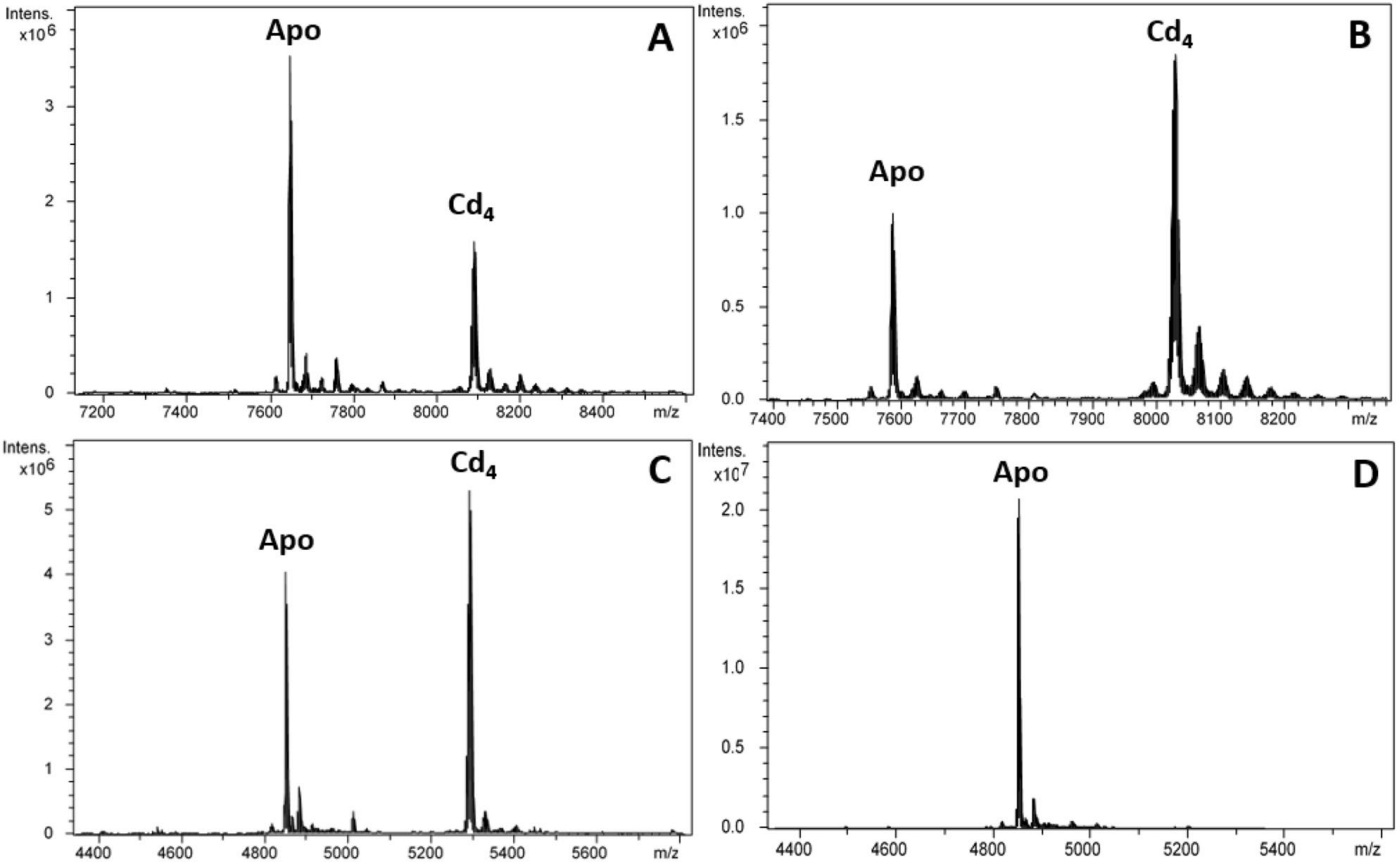
Deconvoluted ESI–MS spectra carried out at pH 2.4 of the Cd(II)-enriched preparations of (**A**) LgiMT1, (**B**) LgiMT2 and (**C**) γLgiMT2 produced in *E.* coli cultures. (**D**) Deconvoluted ESI–MS spectra of the Cd-γLgiMT2 preparation after acidification of the sample at pH 1.0.

**Figure 3:**
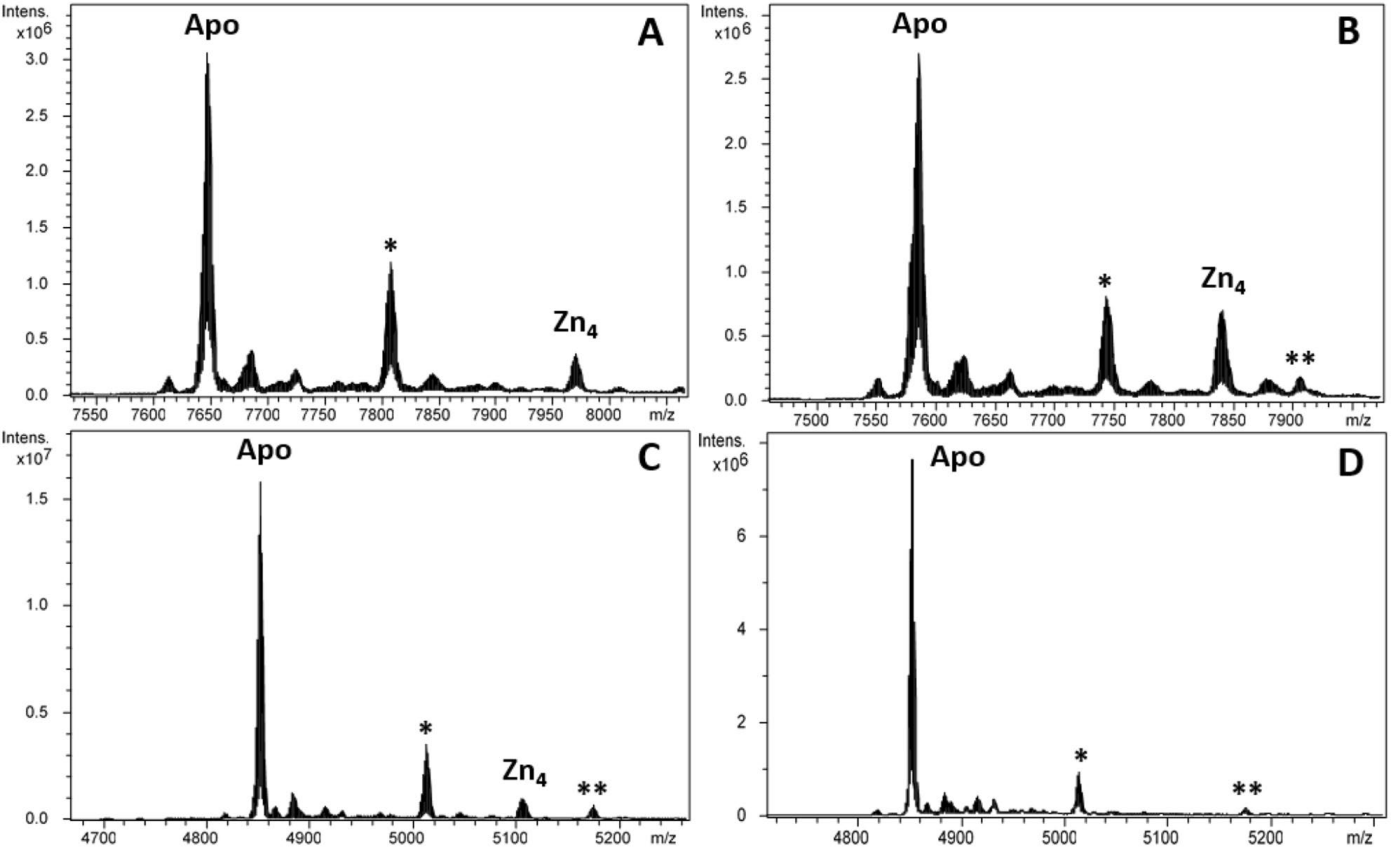
Deconvoluted ESI–MS spectra carried out at pH 2.4 of the Zn(II)-enriched preparations of (**A**) LgiMT1, (**B**) LgiMT2 and (**C**) γLgiMT2 produced in *E. coli* cultures. (**D**) Deconvoluted ESI–MS spectra of the Zn-γLgiMT2 preparation after acidification of the sample at pH 1.0. The asterisk (*) stands for mono-glycosylated apo species and double asterisk (**) stands for di-glycosylated apo species.

To explore the metal-binding features of the two *L. gigantea* MTs as well as of the γ domain, we studied the formation of metal–MT complexes by ESI–MS analyses. LgiMT1, LgiMT2 and the γ domain of LgiMT2 (from Met1 to Gln45; hereafter γLgiMT2) were heterologously expressed in *E. coli* grown in media supplemented with Cu(II), Cd(II) or Zn(II) salts. The election of the γ domain of LgiMT2 relied on the fact that it was more conserved than that of LgiMT1 when compared across the γ domains of other Patellogastropoda MTs (Fig. 1). The analysis of acidic ESI–MS performed with the recombinant LgiMT1 and LgiMT2 isoforms and with the isolated γLgiMT2 domain recovered from Cd(II)- or Zn(II)-enriched cultures confirmed the identity of the recombinantly expressed MTs (Figs. 2 and 3). Their experimental molecular weights (7648.2 Da, 7585.9 Da and 4852.0 Da respectively, for LgiMT1, LgiMT2 and γLgiMT2) precisely match the theoretical values (7648.9 Da, 7586.7 Da and 4852.6 Da). Noteworthy, in all these preparations, Cd_4_-species (major peaks in Fig. 2B and C, and minor in A) or Zn_4_-species (minor peaks in Fig. 3A–C) remained present at pH 2.4, and only lowering the pH to 1.0 allowed their complete demetalation (Figs. 2D and 3D). The reluctance of *L. gigantea* MTs to exchange the divalent metal ions for protons was surprising since it had been never observed before for any other recombinantly produced MT, and it will be discussed below.

**Figure 4:**
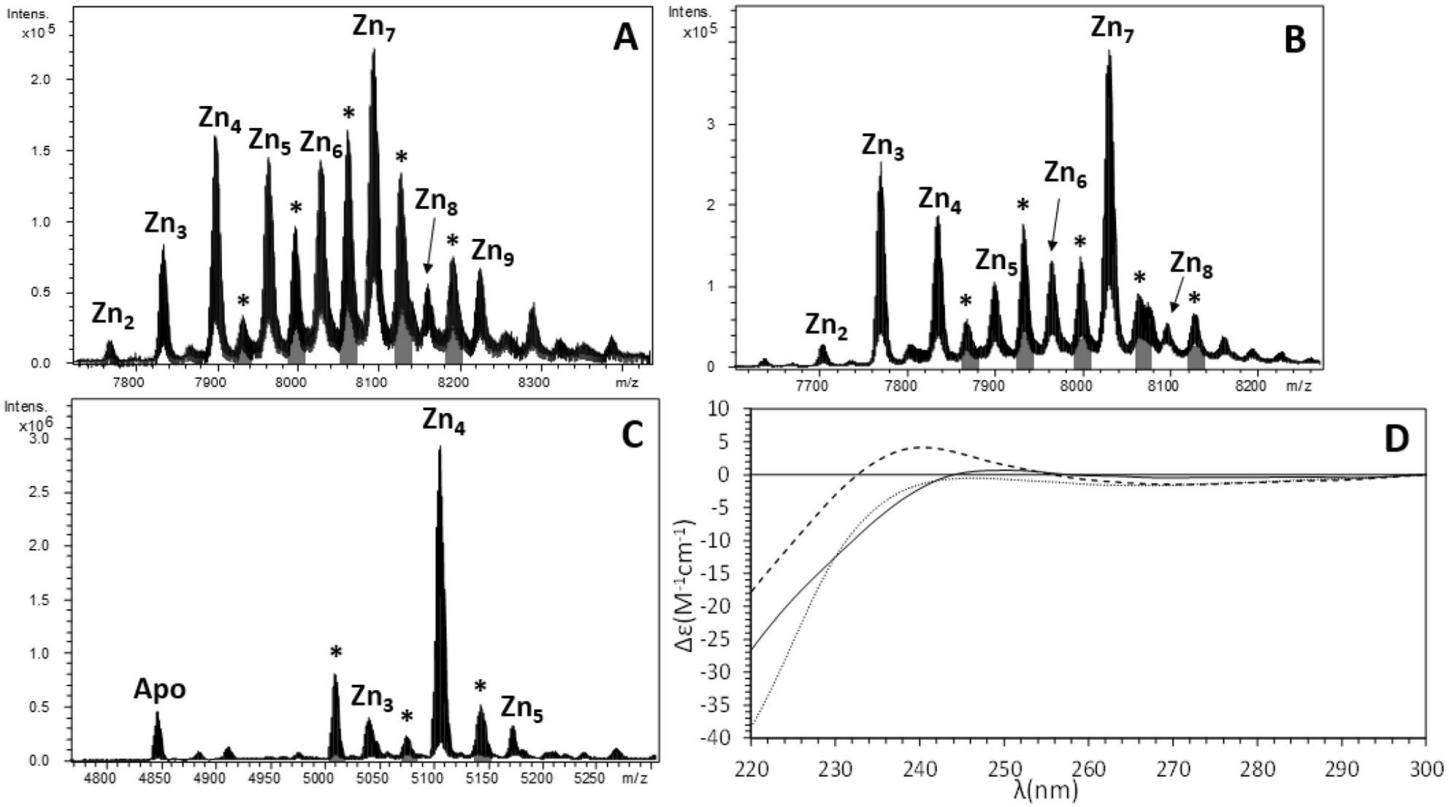
Deconvoluted ESI–MS spectra carried out at pH 7.0 of the Zn(II)-enriched preparations of (**A**) LgiMT1, (**B**) LgiMT2 and (**C**) γLgiMT2 produced in *E. coli* cultures. The asterisk (*) stands for mono-glycosylated Zn-MT species whose peaks have been shaded in gray in the spectra. In (**A**) and (**B**), the Zn-MT* species range from Zn_2_- to Zn_6_-MT*; in (**C**) they correspond to apo*, Zn_1_-MT* and Zn_2_-MT*. (**D**) Superposition of the CD envelopes of the Zn-LgiMT1 (solid line), Zn-LgiMT2 (dashed line) and Zn-γLgiMT2 (pointed line) preparations.

Equally remarkable was the presence in Zn(II)-enriched productions of additional peaks in the ESI–MS spectra at acidic and physiologic pHs (marked with asterisks in Figs. 3 and 4). At pH 1, all Zn(II) ions have been replaced by protons, but the spectra still showed two extra peaks (Fig. 3D), one with an additional mass of 162 Da (marked with *) and another with two additional masses of 162 Da (marked with **), suggesting that one or two monomers of 162 Da might be covalently attached to the MT. We extensively investigated this modification elsewhere demonstrating that they represent glycosylated forms that appear when MTs are recombinantly produced with the non-cognate metal ions Zn(II) and Cu(I)^32^. In contrast, glycosylation is absent when MTs are produced in the presence of their cognate metal, which is most likely the functionally relevant one, meaning that glycosylation does not alter the results about the stability or the metal-affinity of recombinantly produced MTs.

### LgiMT1 and LgiMT2 bind divalent metal ions with a high preference for Cd(II)

**Figure 5:**
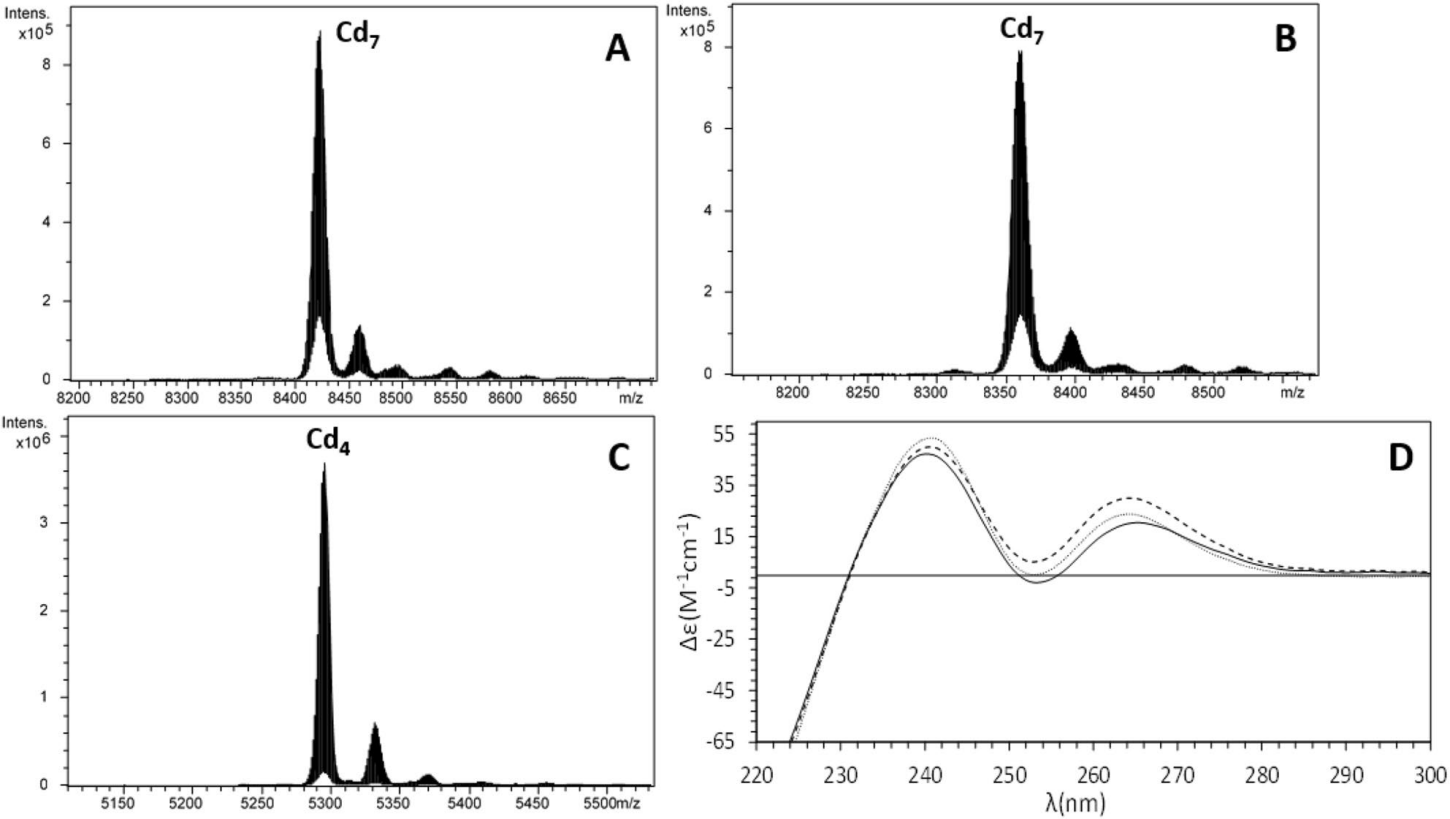
ESI–MS spectra at pH 7.0 of the Cd-LgiMT1 (**A**), Cd-LgiMT2 (**B**) and Cd-γLgiMT2 (**C**) recombinant preparations. All minor peaks showed in the ESI–MS spectra correspond to the ionization of the single major species with Na^+^ ions (sodium adducts). (**D**) Superposition of the CD envelopes of the Cd-LgiMT1 (solid line), Cd-LgiMT2 (dashed line) and Cd-γLgiMT2 (pointed line) preparations.

**Figure 6:**
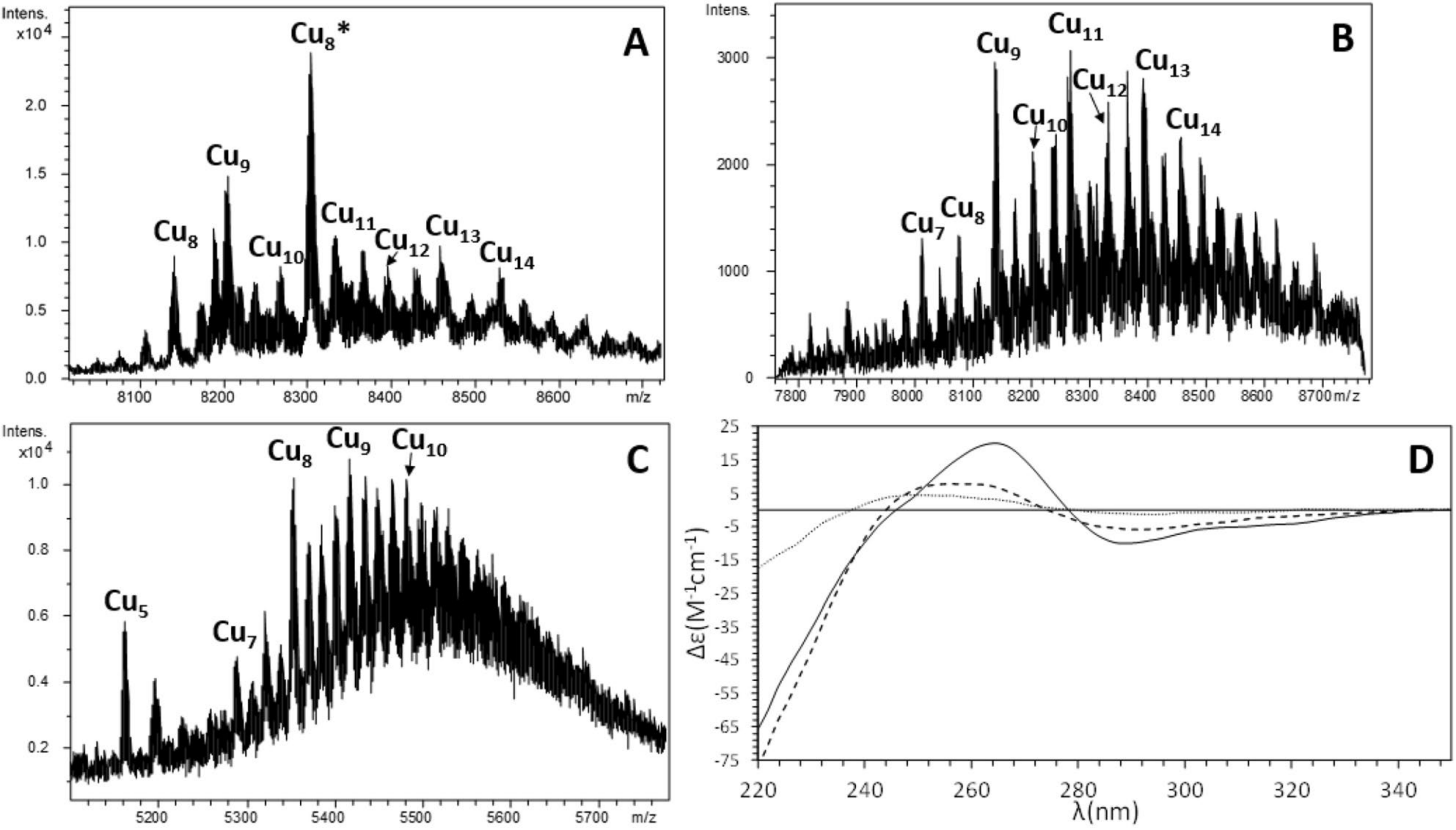
ESI–MS spectra at pH 7.0 of the Cu-LgiMT1 (**A**), Cu-LgiMT2 (**B**) and Cu-γLgiMT2 (**C**) recombinant preparations. (**D**) Superposition of the CD envelopes of the Cu-LgiMT1 (solid line), Cu-LgiMT2 (dashed line) and Cu-γLgiMT2 (pointed line) preparations. The asterisk (*) stands for a Cu_8_-LgiMT1 mono-glycosylated species.

In order to characterize the biochemical properties and metal-binding features of LgiMT1 and LgiMT2, we studied the formation of metal–MT complexes by ESI–MS analyses when they were produced under Cd, Zn or Cu surplus conditions. Notice that the metal-MT complexes of LgiMT1 and LgiMT2 productions enriched with the same metal displayed almost the same speciation by ESI–MS (e.g., compare panels A and B in Fig. 4 or in Fig. 5) probably due to the high degree of sequence identity between both isoforms (Fig. 1B). In contrast, the metalated samples for both MTs obtained with different metals, i.e. under Cd-, Zn- or Cu-enriched media, were clearly different (e.g., compare panels A from Figs. 4, 5 and 6), suggesting a clear metal preference. In Cd surplus conditions, Cd_7_-LgiMT1 and Cd_7_-LgiMT2 were the only species detected by ESI–MS under physiological pH conditions (Fig. 5A and B), in agreement with the ICP-AES results (7.3 Cd/LgiMT1 and 7.7 Cd/LgiMT2). Additionally, Cd_7_-LgiMTs’ metal clusters showed very similar structuration, both isoforms rendering equally intense analogous CD envelopes (Fig. 5D). It was remarkable, from the envelopes, that an exciton coupling at *ca.* 250 nm, characteristic of Cd(SCys)_4_ chromophores, seemed to appear (confirming a high structuration degree of the chromophores), even if the negative band of the exciton coupling has disappeared by the very high intensity of the band related with the secondary sequence of the protein and the peptide bonds (up to 240 nm). These spectroscopic fingerprints also contrasted with those obtained for the Zn-preparations (Fig. 4D) that were of very low intensity and almost featureless. Zn fingerprints were, indeed, in nice correspondence with the mixture of Zn-MT complexes, ranging from Zn_2_- to Zn_9_-MT, obtained when LgiMTs were expressed in Zn surplus conditions (Fig. 4). These results suggested that LgiMTs are not ‘genuine’ Zn-thioneins, which typically yield a unique and well-structured species when synthesized in Zn-enriched media^4^, but they show a strong Cd-thionein character. This character was additionally supported by the presence of glycosylated MT species in the productions under Zn surplus conditions (Fig. 3), which is characteristic of partly structured Cd-thioneins when produced in absence of their preferred metal^32^.

Finally, the productions of LgiMTs in Cu(II) surplus conditions also sustained their Cd-specificity. ‘Genuine’ Cu-thioneins render single homometallic species when synthesized under Cu surplus but yield a mixture of species when produced in Cd(II)- or Zn(II)-enriched cultures^4^. These mixtures are of homometallic Cu-MT complexes for Cd-thioneins, and of heterometallic Zn/Cu-MT species for Zn-thioneins^33^. Our results clearly showed a mixture of homometallic Cu-MT complexes (12.1 Cu/LgiMT1 and 14.3 Cu/LgiMT2), confirming the Cd-thionein character of both LgiMTs (Fig. 6). Spectroscopically, these mixtures display a faint CD envelope, with only Cu-LgiMT1 exhibiting a slightly higher intensity, although the differences between the samples are barely noticeable in the CD envelopes of both homologues (Fig. 6D). As observed by ESI–MS, LgiMT2 displayed monomers and dimers with equally intense peaks, contrasting with the Cu-LgiMT1 sample, whose major peak corresponds to a glycosylated Cu_8_-MT complex. As explained above, glycosylation occurs when MTs are obtained with Cu(I) -or Zn(II)- ions bound, but these are not their preferred ones.

### Characterization of the novel γ domain with a suggested adamantane structure

The most conspicuous feature of LgiMTs was their γ domain, a novel MT domain that seems to be exclusive of the Patellogastropoda clade. We characterized the biochemical properties and metal-binding features of this domain from the LgiMT2 isoform. The γLgiMT2 domain rendered a unique Cd_4_-γLgiMT2 species when synthesized under Cd(II) surplus (Fig. 5C), which was still present at pH 2.4 (Fig. 2C). The removal of those 4 Cd(II) metal ions required the acidification of the samples down to pH 1.0 (Fig. 2D), thus highlighting the extremely high affinity of these proteins for Cd(II) and the high degree of cooperativity in the formation of the M^II^_4_(SCys)_10_ clusters, as no intermediate species were detected. Similar results, although less intense, were observed in Zn(II) surplus productions. Zn_4_-LgiMT2 and Zn_4_-γLgiMT2 peaks were still present at pH 2.4 (Fig. 3C), and pH 1.0 was needed to release these bound Zn(II) ions (Fig. 3D). Additionally, the fact that both Cd-LgiMTs and the Cd-γLgiMT2 fragment render the same CD envelope evidences that the Cd_4_(SCys)_10_ metal cluster is spectroscopically dominant over the other half of the entire protein.

**Figure 7:**
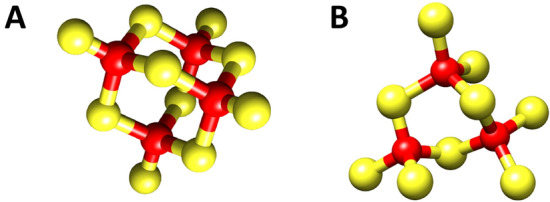
(**A**) The *proposed* novel adamantane M^II^_4_(SCys)_10_ cluster and (**B**) the cyclohexane-like ring structure in the M^II^_3_(SCys)_9_ cluster of a "conventional" gastropod CdMT, as suggested for HpCdMT of the Roman snail *Helix pomatia*^38^.

Overall, our data strongly support the evidence that LgiMTs are modular proteins structured in two domains, a ‘stable’ N-terminal γ domain of 10 Cys reluctant to release its metal ions, and a more ‘labile’ C-terminal β1 domain with 9 Cys that forms a Cd_3_(SCys)_9_ cluster with poor contribution to the final CD envelope, and that easily exchanges metal ions. Our data also suggest that the strong Cd-thionein character of the γ domain is transferred to the full-length LgiMTs proteins. Based on its stoichiometry and a comparison with stoichiometries and structures of three-metal and four-metal clusters from other MTs (Table 1), we propose that the M^II^_4_(SCys)_10_ cluster of the γ domain adopts an “adamantane” cage structure that contrasts with the cyclohexane-like ring structure in the M^II^_3_(SCys)_9_ cluster of the “conventional” gastropod β1 domains (Fig. 7), as first suggested by a spectroscopic study^48^, and recently demonstrated by NMR structure determination and resolved metal connectivities for the HpCdMT of the Roman snail, *Helix pomatia*^24^. An “adamantane” cage structure would not be new in the inorganic field of thiolate metal complexes^34,35^ although, to our knowledge, has never been reported in living organisms and for sure would represent a new structural motif in MTs.

**Table 1:** Cluster structure geometry and topologies in MT domains of different species of molluscs (in bold letters) and vertebrates compared to the proposed structure of the γ domains of *Lottia gigantea* MTs (present study, underline), highlighting the respective cluster stoichiometries, the domains in which they reside, the coordination number of Cd^II^ ions in the complex structure, the geometry of the cluster structure, and references to which the respective data refer.

Species (MT)	Cluster Stoichiometry	Domain (N/C-terminal)	Coordination number	Cluster structure geometry	References
*Rattus rattus* (MT-1)	M^II^_3_(SCys)_9_	β domain (N-terminal)	4	Cyclohexane Ring (“distorted chair”)	^36,37^
***Helix pomatia* (CdMT)**	M^II^_3_(SCys)_9_	β3, β1 domains (N and C-terminal)	4	Cyclohexane Ring (“distorted chair”)	^24,38^
***Lottia gigantean* (LgMT1/LgMT2)**	M^II^_4_(SCys)_10_	γ domain (N-terminal)	4	Adamantane	This study
*Rattus rattus* (MT-1)	M^II^_4_(SCys)_11_	α domain (C-terminal)	4	Boat Conformation	^36,37^
***Mytilus edulis***, ***M. galloprovincialis* (MT-10)**	M^II^_4_(SCys)_12_	α domain (N-terminal)	4	“Distorted Boat” Conformation	^35,39,40^

### The new Patellogastropoda γ domain: an evolutionary resounding and ecologically significant innovation

All limpet MTs so far known show in their primary structure the typical sequence features as described for the two MTs of *Lottia gigantea* (Fig. 1). It can therefore be assumed that the described γ-domain with its suggested adamantane 3D structuration is a lineage-specific evolutionary innovation that applies to all extant Patellogastropoda. At the same time, there is no doubt that the new domain contributes, compared to other mollusk MTs, significantly to the improvement and optimization of the Cd binding performance of limpet MTs in a twofold manner: First, it confers on them an extraordinary Cd-binding specificity as evidenced, for example, by the particularly strong reluctance of the protein domain to release the Cd(II) ions once bound, even under very strong acidic conditions (Figs. 2D and 3D). Second, the new domain structure improves the Cd(II) binding capacity of limpet MTs by increasing their binding stoichiometry from three to four Cd(II) ions per domain moiety, thus differing from the stoichiometry of the β3-β1 domain configuration found in all other gastropod MTs (Fig. 1). The high Cd specificity at the cost of Zn of limpet MTs is confirmed by previous studies and experiments in vivo^41–43^. It is thus not surprising that limpets are among the marine invertebrates with the reportedly highest accumulation capacity for cadmium^44^, thus having repeatedly been suggested as suitable bioindicators for marine Cd pollution^46,47^. We would like to conclude by stressing that the particular ability of limpets for Cd detoxification by binding the metal to Cd-specific MTs is an ancient feature found in many gastropod lineages and species, evolved through geological eras of volcanic metal emissions^48^. This ability has apparently been optimized in species of the clade of Patellogastropoda such as *Lottia gigantea* (present study) and other limpets, some of them still thriving in marine realms dominated by Cd emanations through volcanic and hydrothermal activities^49^, or in habitats contaminated by anthropogenic Cd emissions^50^.

The discovery of the new γ domain in limpet MTs will pave the way for further investigations focusing, among others, on the 3D structure of this novel domain with its M^II^_4_(SCys)_10_ cluster, and on its affinity constants for divalent metal ions such as Zn(II) and Cd(II).

## Data Availability

All data generated or analyzed during this study are included in this published article.
